# The multidimensionality of soil macroecology

**DOI:** 10.1111/geb.13211

**Published:** 2020-11-11

**Authors:** Nico Eisenhauer, François Buscot, Anna Heintz-Buschart, Stephanie D. Jurburg, Kirsten Küsel, Johannes Sikorski, Hans-Jörg Vogel, Carlos A. Guerra

**Affiliations:** 1 German Centre for Integrative Biodiversity Research (iDiv) Halle-Jena-Leipzig, Leipzig, Germany; 2 Institute of Biology, Leipzig University, Leipzig, Germany; 3 Department of Soil Ecology, Helmholtz Centre for Environmental Research – UFZ, Halle, Germany; 4 Institute of Biodiversity, Friedrich Schiller University Jena, Jena, Germany; 5 Leibniz-Institut DSMZ-Deutsche Sammlung von Mikroorganismen und Zellkulturen, Braunschweig, Germany; 6 Department of Soil System Science, Helmholtz Centre for Environmental Research – UFZ, Halle, Germany; 7 Institute of Biology, Martin Luther University Halle Wittenberg, Halle (Saale), Germany

**Keywords:** biodiversity change, biogeography, ecosystem functioning, environmental change, soil biodiversity

## Abstract

The recent past has seen a tremendous surge in soil macroecological studies and new insights into the global drivers of one-quarter of the biodiversity of the Earth. Building on these important developments, a recent paper in *Global Ecology and Biogeography* outlined promising methods and approaches to advance soil macroecology. Among other recommendations, White and colleagues introduced the concept of a spatial three-dimensionality in soil macroecology by considering the different spheres of influence and scales, as soil organism size ranges vary from bacteria to macro- and megafauna. Here, we extend this concept by discussing three additional dimensions (biological, physical, and societal) that are crucial to steer soil macroecology from pattern description towards better mechanistic understanding. In our view, these are the requirements to establish it as a predictive science that can inform policy about relevant nature and management conservation actions. We highlight the need to explore temporal dynamics of soil biodiversity and functions across multiple temporal scales, integrating different facets of biodiversity (i.e., variability in body size, life-history traits, species identities, and groups of taxa) and their relationships to multiple ecosystem functions, in addition to the feedback effects between humans and soil biodiversity. We also argue that future research needs to consider effective soil conservation policy and management in combination with higher awareness of the contributions of soil-based nature’s contributions to people. To verify causal relationships, soil macroecology should be paired with local and globally distributed experiments. The present paper expands the multidimensional perspective on soil macroecology to guide future research contents and funding. We recommend considering these multiple dimensions in projected global soil biodiversity monitoring initiatives.

Biodiversity is changing around the globe in response to naturally and anthropogenically driven environmental changes (Díaz et al., 2019; IPBES, 2019). Human activities have caused exceptionally high rates of decline in biodiversity across the world, threatening the integrity and functioning of these ecosystems and the services they provide to society (Cardinale et al., 2012; Díaz et al., 2018). To fight the biodiversity crisis, knowledge about the main drivers of biodiversity, their context dependence, and their future impacts has to be generated and implemented in conservation actions. However, for many taxa, we currently lack such knowledge. One crucial example is soil biodiversity which encompasses roughly one-quarter of all species on Earth, with high relevance for ecosystem functioning (Bardgett & van der Putten, 2014) and ecosystem service provisioning (Wall et al., 2015). This massive knowledge gap has generated a recent surge in global soil biodiversity syntheses, with several soil macroecological analyses based on observation networks (e.g., Crowther et al., 2019) and meta-analyses of experimental results (e.g., Zhou et al., 2020). These efforts have recently produced new insights into the global distribution of several soil taxa (e.g., Delgado-Baquerizo et al., 2018; Oliverio et al., 2020; Phillips et al., 2019; van den Hoogen et al., 2019; Figure 1a) and highlighted substantial differences in distribution from aboveground biodiversity that currently informs most conservation actions (e.g., Cameron et al., 2019; Tedersoo et al., 2014), but also the significance of spatial scale for aboveground–belowground biodiversity comparisons (Phillips et al., 2019).

In addition to repeated calls to address major global sampling and data blind spots in soil macroecology (Cameron et al., 2018; Guerra et al., 2020), White and colleagues (2020) recently outlined promising methods and approaches to advance soil macroecology. Beside the need to expand the classical macroecological toolbox, to consider new sampling techniques, molecular identification, functional approaches, environmental variables, different temporal scales and modelling techniques, they introduce the need to account for the three-dimensionality of soil macroecology by also considering the different soil depths that soil organisms inhabit (Figure 1b). This is supported by the assumption that the drivers and distribution patterns of both soil biodiversity and ecosystem functions might depend on soil depth (Eisenhauer et al., 2018). Moreover, they propose that the different spheres of influence and spatial scales that play a role in soil macroecology should be taken into account, because soil organisms, which range from bacteria to macro- and megafauna, can have substantially different zones and times of activity and influence. For example, anoxic conditions in deeper soils can cause exclusion of meso- to macroscopic eukaryotes and associated physical and/or ecological traits, leading to the preservation and accumulation of organic material, which affects soil carbon storage capacity (Beulig et al., 2016). A promising step forward would be the development of multiscale sampling protocols (Rasmussen et al., 2018) that consider “scales of effect” and local spatial compartmentalization (Thakur et al., 2020). Support for these claims comes from empirical work showing that the depth distribution and major ecosystem effects of soil organisms differ among soil layers, as exemplified by dissimilar effects of arbuscular mycorrhizal fungi and ectomycorrhizal fungi on carbon sequestration along the soil profile (Craig et al., 2018). Scientists have started to go even deeper and extend soil biodiversity research to the subsoil, where strong signals of aboveground biodiversity and management can still be detected (Küsel et al., 2016).

We build on the recent paper by White et al. (2020) and propose that soil macroecology should be expanded to essential biological, physical, and societal dimensions that can open the fields of soil macroecology, global change ecology, and interaction ecology to a new, more insightful understanding of soil systems. First, soil macroecology has to consider the variability of soil biodiversity and functions over time (Figure 1c). Although some soil taxa are now considered on International Union for Conservation of Nature (IUCN) red lists, there is almost no information on temporal trends for these and other soil taxa (Eisenhauer et al., 2019; Phillips et al., 2017). Data syntheses based on snapshot assessments of soil biodiversity and functions are a crucial first step to gain basic information on relevant drivers and global gradients. Yet, such assessments have limited capacity to inform policymakers about temporal trends and consequences of biodiversity change, hampering our ability to identify vulnerable taxa and ecosystems. Although synthesis approaches can allow for the successful integration of research with disparate sampling schemes (i.e., Phillips et al., 2019; van Klink et al., 2020), standardized samplings across appropriate time-scales (e.g., every 2–3 years, ideally for ≥10 years) will allow us to understand and predict changes in soil biodiversity and function distribution better (Guerra et al., 2020).

Moreover, it is well known that soil organisms show strong responses to recurring environmental changes, such as seasonal dynamics (Žifčáková et al., 2017), recurring fire regimens (Oliver et al., 2015), and freeze–thaw cycles, in their population sizes and activity (Eisenhauer et al., 2018). These temporal dynamics are important for soil habitats and are a special feature of them, and they might be key to understanding the observed biodiversity (White et al., 2020). For example, from the perspective of soil organisms, the supply of resources is not a constantly dripping source but is highly episodic, forcing them into inactive and dormant stages for most of their lifetime (Blagodatskaya & Kuzyakov, 2013; White et al., 2020). Besides this, changes in soil moisture impact fluxes of water and matter, in addition to gas diffusivities, leading to highly dynamic redox conditions and fundamental changes in the microbial metabolism (Smith et al., 2003). Furthermore, small-scale heterogeneity in the soil matrix, which conditions access to resources (Bickel & Or, 2020), might provide a chance for the persistence of organisms with low competitiveness (Portell et al., 2018) and maintain crucial functions, such as carbon cycling, running in modified conditions (Banerjee et al., 2016). The amplitude of these dynamics, together with temperature as another crucial control of biological activity, is very high close to the soil surface and is dampened significantly with increasing soil depth. This is another reason why considering the spatial and temporal dimensions is crucial for a proper assessment of soil conditions.

Gradual but persistent environmental changes, such as climate change, might also cause shifts in the activity patterns and phenology of soil organisms (Siebert et al. 2019; Thakur et al., 2018). Recurring and persistent environmental change can have complex effects on soil biota, resulting in modified resilience to further perturbations (Knox et al., 2017), but with unknown consequences for the phenology of aboveground–belowground species interactions and ecosystem functioning (Eisenhauer et al., 2018). Therefore, not accounting for these temporal dynamics not only limits our ability to understand soil systems, but also to harness their benefits to society (i.e., soil ecosystem services; Bach et al., 2020). Two recently announced, complementary monitoring activities might allow us to gain urgently needed information about long-term trends and short-term dynamics, respectively. The soil biodiversity observation network, Soil BON (https://geobon.org/bons/thematic-bon/soil-bon/), is planning to perform repeated global assessments every 3 years (Figure 1c-i), and the Lifeplan project (https://www.helsinki.fi/en/projects/lifeplan/about) will explore within-year variability in aboveground and belowground biodiversity activity for multiple years (Figure 1c-ii). Ultimately, it will be important to compare such macroecological time-series data for aboveground and belowground organisms.

Another important temporal aspect of the distribution of soil biodiversity is soil history. Many recent biodiversity trend analyses lack crucial baseline information on previous land cover and local biodiversity drivers (Eisenhauer et al., 2016). For instance, pedogenesis is a crucial factor for soil biodiversity (Delgado-Baquerizo et al., 2019), and past land uses can have long-lasting effects on soil abiotic and biotic properties (e.g., Bachelot et al., 2016; Demetrio et al., 2019). More fundamentally, pedogenesis, as a mechanism of soil formation that is largely driven biologically, is paralleled by gradual disbalances in the forms and availabilities of key resources, such as nitrogen and phosphorus. This strongly impacts on the dynamics of soil organism communities (Turner & Condron, 2013), and soil management has an impact on these dynamics (Chen et al., 2015). Soil organic carbon dynamics and their responses to climatic control or land use are strongly dependent on soil depth (Balesdent et al., 2018). Given that about half of this carbon is located below 30 cm depth, decision-makers and ecosystem managers need a better data basis for the management of the deep carbon stocks. Human activities, such as the addition of fertilizer, leguminous crop production, and combustion processes, have also altered the nitrogen budget in deep soils, with unknown consequences for soil ecosystem functioning. There is evidence that even in sub-soils the carbon dynamics are largely controlled by biological processes (Hobley et al., 2017). Finally, soil biodiversity affects soil erosion rates (Orgiazzi & Panagos, 2018) and vice versa (Guerra, Rosa, et al., 2020). Thus, geological history and past and present human activities alter biodiversity and its distribution, which is why we encourage researchers to consider such important effects of soil history that act on different spatial and temporal scales. This can be done by explicitly considering different soil histories in designing soil biodiversity monitoring schemes and by integrating the respective information in data analysis (Delgado-Baquerizo et al., 2020).

Beyond studying the spatial and temporal distribution of certain representatives of soil biodiversity, the varying vulnerability of soil organisms to different environmental challenges needs to be addressed (Bastida et al., 2020; Coyle et al., 2017; Figure 1d-i). Given the variability of known responses, these taxa should also represent different body sizes, life-history strategies, and functional and trophic groups (Blankinship et al., 2011). Building on that knowledge, a soil food web perspective will allow inferences to be made about important linkages among taxa and, thus, an understanding of the joint or dissimilar environmental drivers for different taxa (Figure 1d-ii). Moreover, different facets of biodiversity can be representative of changes in soil biodiversity and of relationships with multiple ecosystem functions (Delgado-Baquerizo et al., 2020; Figure 1d-iii). For instance, a recent meta-analysis showed that the effects of different environmental stressors on soil biodiversity vary between biodiversity facets (species richness and population density of decomposers) and that these changes can have significant ecosystem consequences (Beaumelle et al., 2020). A multitaxa and multitrophic perspective (Soliveres et al., 2016) might require collaboration between a broad range of soil ecologists and taxonomists and the integration of molecular with classical count data (Guerra et al., 2020; White et al., 2020). This research is particularly relevant, because common macroecological rules might not apply to soil organisms (e.g., Cameron et al., 2019; Frelich et al., 2012; Phillips et al., 2019), highlighting the need for soil macroecological theory to be developed (Eisenhauer et al., 2017) by considering the distribution of soil biodiversity across different spatial and temporal scales (Phillips et al., 2019; Thakur et al., 2020).

Finally, we suggest that the human dimension of soil biodiversity distribution should be considered by accounting for the feedback effects between society and soil biodiversity (Figure 1e). On the one hand, multiple soil ecosystem functions deliver important nature’s contributions to people (Díaz et al., 2018; Geisen et al., 2019). On the other hand, human activities influence soil biodiversity (Tsiafouli et al., 2015; Wall et al., 2015), and adequate environmental policies will determine the conservation and fate of soil biodiversity (Bach et al., 2020). Human impacts on soils can last for a long time (e.g., Demetrio et al., 2019) and reach deep, influencing biogeochemical cycles in the subsurface and the quality of subterranean water (e.g., Küsel et al., 2016). Soil ecologists need to step up to gather and provide the relevant data to inform biodiversity reports (e.g., IPBES, 2019) and strategies on how to manage soil biodiversity in a sustainable way (Geisen et al., 2019; Wall et al., 2015). To be effective mechanisms of soil conservation, these strategies need to overcome the use of locally specific indicators to rely on measurements that can be communicated across ecosystem and political boundaries. The development of widely accepted indicators for soil biodiversity and soil health with global relevance would thus be an essential step forward (Schloter et al., 2018). Moreover, we need to start to appreciate the extrinsic and intrinsic value of soil organisms and to bring soil biodiversity into the public discourse (Phillips et al., 2020).

Overall, by adding the dimensions of time, different facets of biodiversity, linkages to ecosystem functioning, and reciprocal relationships between soil biodiversity and society, we aim to extend the work by White et al. (2020). We outline how soil macroecology could work beyond the description of patterns and have a direct impact on our understanding of global ecological and biogeochemical processes (Crowther et al., 2019). Such a process-based understanding would benefit from the pairing of soil macroecological studies with local and globally distributed experiments that have great potential to inform each other. For instance, a recent macroecological study on soil pathogens of plants used complementary data from a field experiment to test the relevance of temperature (Delgado-Baquerizo et al., 2020). Moreover, globally distributed experimental networks, such as Nutrient Network (Borer et al., 2014) and Drought-Net (https://drought-net.colostate.edu/), can link macroecological patterns with potential context-dependent effects of global change drivers. More than a theoretical exercise, these steps could have real-world implications by producing more adequate assessments of the impacts of global change, improving the predictive modelling at multiple scales, particularly of soil communities and functions, and informing both management (at local scales) and policymaking (at broader scales). Therefore, accounting for the multidimensionality of soil macroecology does not necessarily translate into increasing the complexity of our responses, but into a better understanding of soil ecology and more informed decision-making.

## Figures and Tables

**Figure 1:**
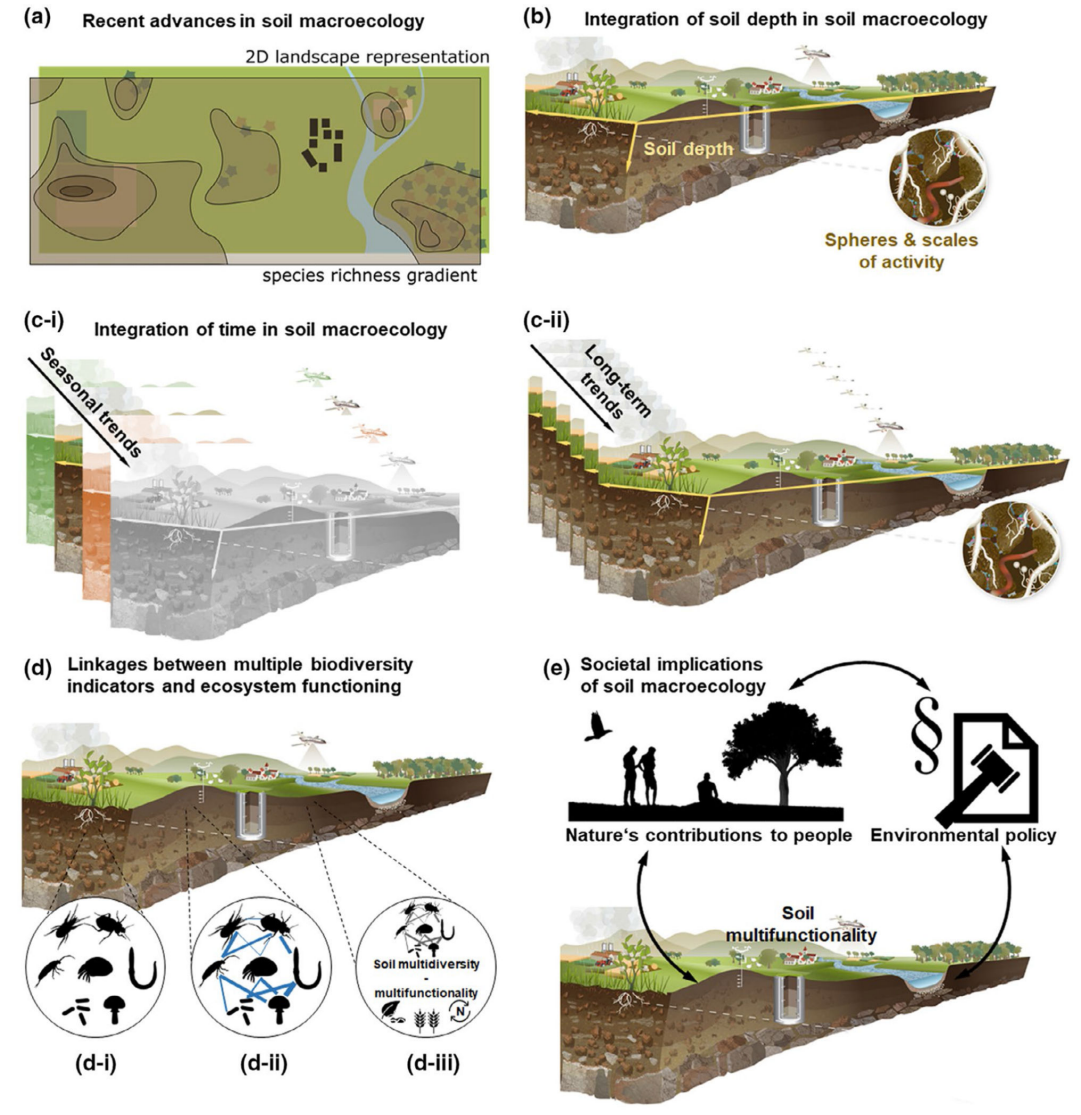
Multidimensionality in soil macroecology. (a) Soil macroecology has been lagging behind the macroecology of aboveground taxa. However, there have been recent advances in soil macroecology based on global surveys and data syntheses. (b) White et al. (2020) proposed that soil macroecology should be advanced by a consideration of the different soil depths that soil organisms inhabit, because drivers and distribution patterns might depend on soil depth. Moreover, they proposed that the different spheres and spatial scales that play a role in soil macroecology should be taken into account, because soil organisms range from bacteria to macro- and megafauna that might have substantially different zones of activity and influence. (c) We propose that soil macroecology should be expanded to a fourth dimension by considering the variability of soil biodiversity and functions over time. (c-i) It is well known that soil organisms show strong seasonal dynamics in their population sizes and activity. (c-ii) Snapshot assessments of soil biodiversity and functions are a crucial first step to gain basic information on relevant drivers and global gradients. However, such assessments lack information on crucial temporal trends of biodiversity change. Only samplings across multiple years will allow us to understand and predict changes in soil biodiversity and distribution of functions. (d-i) Beyond studying the spatial and temporal distribution of certain representatives of soil biodiversity, different taxa need to be studied, representing different size and trophic groups. (d-ii) Building on that, a soil food web perspective will allow inference concerning important linkages among taxa and, thus, an understanding of the joint or dissimilar environmental drivers for different taxa. (d-iii) Different facets of biodiversity can be representative of changes in soil biodiversity in addition to relationships with multiple ecosystem functions (depicted examples are litter decomposition, crop production, and nutrient cycling). (e) Moreover, we suggest that the human dimension of the distribution of soil biodiversity should be considered. One the one hand, multiple soil ecosystem functions deliver important nature’s contributions to people. On the other hand, human activities influence soil biodiversity, and adequate environmental policies will determine the conservation and fate of soil biodiversity [Colour figure can be viewed at wileyonlinelibrary.com]

## References

[R1] Bach EM, Ramirez KS, Fraser TD, Wall DH (2020). Soil biodiversity integrates solutions for a sustainable future. Sustainability.

[R2] Bachelot B, Uriarte M, Zimmerman JK, Thompson J, Leff JW, Asiaii A, Koshner J, McGuire K (2016). Long-lasting effects of land use history on soil fungal communities in second-growth tropical rain forests. Ecological Applications.

[R3] Balesdent J, Basile-Doelsch I, Chadoeuf J, Cornu S, Derrien D, Fekiacova Z, Hatté C (2018). Atmosphere–soil carbon transfer as a function of soil depth. Nature.

[R4] Banerjee S, Kirkby CA, Schmutter D, Bissett A, Kirkegaard JA, Richardson AE (2016). Network analysis reveals functional redundancy and keystone taxa amongst bacterial and fungal communities during organic matter decomposition in an arable soil. Soil Biology and Biochemistry.

[R5] Bardgett RD, Van Der Putten WH (2014). Belowground biodiversity and ecosystem functioning. Nature.

[R6] Bastida F, Eldridge DJ, Abades S, Alfaro FD, Gallardo A, García-Velázquez L, García C, Hart SC, Pérez CA, Santos F, Trivedi P (2020). Climatic vulnerabilities and ecological preferences of soil invertebrates across biomes. Molecular Ecology.

[R7] Beaumelle L, De Laender F, Eisenhauer N (2020). Biodiversity mediates the effects of stressors but not nutrients on litter decomposition. eLife.

[R8] Beulig F, Urich T, Nowak M, Trumbore SE, Gleixner G, Gilfillan GD, Fjelland KE, Küsel K (2016). Altered carbon turnover processes and microbiomes in soils under long-term extremely high CO_2_ exposure. Nature Microbiology.

[R9] Bickel S, Or D (2020). Soil bacterial diversity mediated by microscale aqueous-phase processes across biomes. Nature Communications.

[R10] Blagodatskaya E, Kuzyakov Y (2013). Active microorganisms in soil: Critical review of estimation criteria and approaches. Soil Biology and Biochemistry.

[R11] Blankinship JC, Niklaus PA, Hungate BA (2011). A meta-analysis of responses of soil biota to global change. Oecologia.

[R12] Borer ET, Harpole WS, Adler PB, Lind EM, Orrock JL, Seabloom EW, Smith MD (2014). Finding generality in ecology: A model for globally distributed experiments. Methods in Ecology and Evolution.

[R13] Cameron EK, Martins IS, Lavelle P, Mathieu J, Tedersoo L, Bahram M, Gottschall F, Guerra CA, Hines J, Patoine G, Siebert J (2019). Global mismatches in aboveground and belowground biodiversity. Conservation Biology.

[R14] Cameron EK, Martins IS, Lavelle P, Mathieu J, Tedersoo L, Gottschall F, Guerra CA, Hines J, Patoine G, Siebert J, Winter M (2018). Global gaps in soil biodiversity data. Nature Ecology & Evolution.

[R15] Cardinale BJ, Duffy JE, Gonzalez A, Hooper DU, Perrings C, Venail P, Kinzig AP (2012). Biodiversity loss and its impact on humanity. Nature.

[R16] Chen CR, Hou EQ, Condron LM, Bacon G, Esfandbod M, Olley J, Turner BL (2015). Soil phosphorus fractionation and nutrient dynamics along the Cooloola coastal dune chronosequence, southern Queensland, Australia. Geoderma.

[R17] Coyle DR, Nagendra UJ, Taylor MK, Campbell JH, Cunard CE, Joslin AH, Mundepi A, Phillips CA, Callaham MA (2017). Soil fauna responses to natural disturbances, invasive species, and global climate change: Current state of the science and a call to action. Soil Biology and Biochemistry.

[R18] Craig ME, Turner BL, Liang C, Clay K, Johnson DJ, Phillips RP (2018). Tree mycorrhizal type predicts within-site variability in the storage and distribution of soil organic matter. Global Change Biology.

[R19] Crowther TW, van den Hoogen J, Wan J, Mayes MA, Keiser AD, Mo L, Averill C, Maynard DS (2019). The global soil community and its influence on biogeochemistry. Science.

[R20] Delgado-Baquerizo M, Bardgett RD, Vitousek PM, Maestre FT, Williams MA, Eldridge DJ, Lambers H, Neuhauser S, Gallardo A, García-Velázquez L, Sala OE (2019). Changes in belowground biodiversity during ecosystem development.

[R21] Delgado-Baquerizo M, Guerra CA, Cano-Díaz C, Egidi E, Wang J-T, Eisenhauer N, Singh BK, Maestre FT (2020). The proportion of soil-borne pathogens increases with warming at the global scale. Nature Climate Change.

[R22] Delgado-Baquerizo M, Oliverio AM, Brewer TE, Benavent-González A, Eldridge DJ, Bardgett RD, Fierer N (2018). A global atlas of the dominant bacteria found in soil. Science.

[R23] Delgado-Baquerizo M, Reich PB, Bardgett RD, Eldridge DJ, Lambers H, Wardle DA, Reed SC, Plaza C, Png GK, Neuhauser S, Berhe AA (2020). The influence of soil age on ecosystem structure and function across biomes. Nature Communications.

[R24] Delgado-Baquerizo M, Reich PB, Trivedi C, Eldridge DJ, Abades S, Alfaro FD, Bastida F, Berhe AA, Cutler NA, Gallardo A, García-Velázquez L (2020). Multiple elements of soil biodiversity drive ecosystem functions across biomes. Nature Ecology & Evolution.

[R25] Demetrio WC, Conrado AC, Acioli A, Ferreira AC, Bartz ML, James SW, Stanton DW (2019). Anthropogenic soils promote biodiversity in Amazonian rainforests. BioRxiv.

[R26] Díaz S, Pascual U, Stenseke M, Martín-López B, Watson RT, Molnár Z, Polasky S (2018). Assessing nature’s contributions to people. Science.

[R27] Díaz S, Settele J, Brondízio ES, Ngo HT, Agard J, Arneth A, Balvanera P, Brauman KA, Butchart SHM, Chan KMA, Garibaldi LA (2019). Pervasive human-driven decline of life on Earth points to the need for transformative change. Science.

[R28] Díaz S, Settele J, Brondízio ES, Ngo HT, Guèze M, Agard J, Zayas CN, IPBES (2019). Summary for policymakers of the global assessment report on biodiversity and ecosystem services of the Intergovernmental Science-Policy Platform on Biodiversity and Ecosystem Services. IPBES secretariat.

[R29] Eisenhauer N, Antunes PM, Bennett AE, Birkhofer K, Bissett A, Bowker MA, Caruso T, Chen B, Coleman DC, Boer WD, Ruiter PD (2017). Priorities for research in soil ecology. Pedobiologia.

[R30] Eisenhauer N, Barnes AD, Cesarz S, Craven D, Ferlian O, Gottschall F, Hines J, Sendek A, Siebert J, Thakur MP, Türke M (2016). Biodiversity–ecosystem function experiments reveal the mechanisms underlying the consequences of biodiversity change in real world ecosystems. Journal of Vegetation Science.

[R31] Eisenhauer N, Bonn A, Guerra CA (2019). Recognizing the quiet extinction of invertebrates. Nature Communications.

[R32] Eisenhauer N, Herrmann S, Hines J, Buscot F, Siebert J, Thakur MP (2018). The dark side of animal phenology. Trends in Ecology and Evolution.

[R33] Frelich LE, Peterson RO, Dovčiak M, Reich PB, Vucetich JA, Eisenhauer N (2012). Trophic cascades, invasive species and bodysize hierarchies interactively modulate climate change responses of ecotonal temperate–boreal forest. Philosophical Transactions of the Royal Society B: Biological Sciences.

[R34] Geisen S, Wall DH, van der Putten WH (2019). Challenges and opportunities for soil biodiversity in the Anthropocene. Current Biology.

[R35] Guerra CA, Heintz-Buschart A, Sikorski J, Chatzinotas A, Guerrero-Ramírez N, Cesarz S, Buscot F (2020). Blind spots in global soil biodiversity and ecosystem function research. Nature Communications.

[R36] Guerra CA, Rosa IM, Valentini E, Wolf F, Filipponi F, Karger DN, Xuan AN, Mathieu J, Lavelle P, Eisenhauer N (2020). Global vulnerability of soil ecosystems to erosion. Landscape Ecology.

[R37] Hobley E, Baldock J, Hua Q, Wilson B (2017). Land-use contrasts reveal instability of subsoil organic carbon. Global Change Biology.

[R38] Knox MA, Andriuzzi WS, Buelow HN, Takacs-Vesbach C, Adams BJ, Wall DH (2017). Decoupled responses of soil bacteria and their invertebrate consumer to warming, but not freeze–thaw cycles, in the Antarctic Dry Valleys. Ecology Letters.

[R39] Küsel K, Totsche KU, Trumbore SE, Lehmann R, Steinhäuser C, Herrmann M (2016). How deep can surface signals be traced in the critical zone? Merging biodiversity with biogeochemistry research in a central German Muschelkalk landscape. Frontiers in Earth Science.

[R40] Oliver AK, Callaham MA, Jumpponen A (2015). Soil fungal communities respond compositionally to recurring frequent prescribed burning in a managed southeastern US forest ecosystem. Forest Ecology and Management.

[R41] Oliverio AM, Geisen S, Delgado-Baquerizo M, Maestre FT, Turner BL, Fierer N (2020). The global-scale distributions of soil protists and their contributions to belowground systems. Science Advances.

[R42] Orgiazzi A, Panagos P (2018). Soil biodiversity and soil erosion: It is time to get married: Adding an earthworm factor to soil erosion modelling. Global Ecology and Biogeography.

[R43] Phillips HR, Beaumelle L, Eisenhauer N, Hines J, Smith LC (2020). Lessons from the WBF2020: Extrinsic and intrinsic value of soil organisms. Soil Organisms.

[R44] Phillips HR, Cameron EK, Ferlian O, Türke M, Winter M, Eisenhauer N (2017). Red list of a black box. Nature Ecology & Evolution.

[R45] Phillips HR, Guerra CA, Bartz ML, Briones MJ, Brown G, Crowther TW, Orgiazzi A (2019). Global distribution of earthworm diversity. Science.

[R46] Portell X, Pot V, Garnier P, Otten W, Baveye PC (2018). Microscale heterogeneity of the spatial distribution of organic matter can promote bacterial biodiversity in soils: Insights from computer simulations. Frontiers in Microbiology.

[R47] Rasmussen PU, Hugerth LW, Blanchet FG, Andersson AF, Lindahl BD, Tack AJ (2018). Multiscale patterns and drivers of arbuscular mycorrhizal fungal communities in the roots and root-associated soil of a wild perennial herb. New Phytologist.

[R48] Schloter M, Nannipieri P, Sørensen SJ, van Elsas JD (2018). Microbial indicators for soil quality. Biology and Fertility of Soils.

[R49] Siebert J, Thakur MP, Reitz T, Schädler M, Schulz E, Yin R, Eisenhauer N (2019). Extensive grassland-use sustains high levels of soil biological activity, but does not alleviate detrimental climate change effects. Advances in Ecological Research.

[R50] Smith KA, Ball T, Conen F, Dobbie KE, Massheder J, Rey A (2003). Exchange of greenhouse gases between soil and atmosphere: Interactions of soil physical factors and biological processes. European Journal of Soil Science.

[R51] Soliveres S, Van Der Plas F, Manning P, Prati D, Gossner MM, Renner SC, Birkhofer K (2016). Biodiversity at multiple trophic levels is needed for ecosystem multifunctionality. Nature.

[R52] Tedersoo L, Bahram M, Põlme S, Kõljalg U, Yorou NS, Wijesundera R, Ruiz LV, Vasco-Palacios AM, Thu PQ, Suija A, Smith ME (2014). Global diversity and geography of soil fungi. Science.

[R53] Thakur MP, Phillips HRP, Brose U, De Vries FT, Lavelle P, Loreau M, Mathieu J, Mulder C, Van der Putten WH, Rillig MC, Wardle DA (2020). Towards an integrative understanding of soil biodiversity. Biological Reviews.

[R54] Thakur MP, Reich PB, Hobbie SE, Stefanski A, Rich R, Rice KE, Eddy WC, Eisenhauer N (2018). Reduced feeding activity of soil detritivores under warmer and drier conditions. Nature Climate Change.

[R55] Tsiafouli MA, Thébault E, Sgardelis SP, de Ruiter PC, van der Putten WH, Birkhofer K, Hemerik L, de Vries FT, Bardgett RD, Brady MV, Bjornlund L (2015). Intensive agriculture reduces soil biodiversity across Europe. Global Change Biology.

[R56] Turner BL, Condron LM (2013). Pedogenesis, nutrient dynamics, and ecosystem development: the legacy of T.W. Walker and J.K. Syers. Plant and Soil.

[R57] Van Den Hoogen J, Geisen S, Routh D, Ferris H, Traunspurger W, Wardle DA, Bardgett RD (2019). Soil nematode abundance and functional group composition at a global scale. Nature.

[R58] van Klink R, Bowler DE, Gongalsky KB, Swengel AB, Gentile A, Chase JM (2020). Meta-analysis reveals declines in terrestrial but increases in freshwater insect abundances. Science.

[R59] Wall DH, Nielsen UN, Six J (2015). Soil biodiversity and human health. Nature.

[R60] White HJ, León-Sánchez L, Burton VJ, Cameron EK, Caruso T, Cunha L, Dirilgen T, Jurburg SD, Kelly R, Kumaresan D, Ochoa-Hueso R (2020). Methods and approaches to advance soil macroecology. Global Ecology and Biogeography.

[R61] Zhou Z, Wang C, Luo Y (2020). Meta-analysis of the impacts of global change factors on soil microbial diversity and functionality. Nature Communications.

[R62] Žifčáková L, Větrovský T, Lombard V, Henrissat B, Howe A, Baldrian P (2017). Feed in summer, rest in winter: Microbial carbon utilization in forest topsoil. Microbiome.

